# Seafood intake and the development of obesity, insulin resistance and type 2 diabetes

**DOI:** 10.1017/S0954422418000240

**Published:** 2019-06

**Authors:** Bjørn Liaset, Jannike Øyen, Hélène Jacques, Karsten Kristiansen, Lise Madsen

**Affiliations:** 1 Institute of Marine Research, PO Box 1870 Nordnes, NO-5817 Bergen, Norway; 2 School of Nutrition, Université Laval, Quebec City, QC G1V 0A6, Canada; 3 Department of Biology, University of Copenhagen, 2200 Copenhagen, Denmark

**Keywords:** Seafood, Fish, Obesity, Type 2 diabetes, Marine PUFA, Body-weight regulation, Glucose regulation

## Abstract

We provide an overview of studies on seafood intake in relation to obesity, insulin resistance and type 2 diabetes. Overweight and obesity development is for most individuals the result of years of positive energy balance. Evidence from intervention trials and animal studies suggests that frequent intake of lean seafood, as compared with intake of terrestrial meats, reduces energy intake by 4–9 %, sufficient to prevent a positive energy balance and obesity. At equal energy intake, lean seafood reduces fasting and postprandial risk markers of insulin resistance, and improves insulin sensitivity in insulin-resistant adults. Energy restriction combined with intake of lean and fatty seafood seems to increase weight loss. Marine *n*-3 PUFA are probably of importance through *n*-3 PUFA-derived lipid mediators such as endocannabinoids and oxylipins, but other constituents of seafood such as the fish protein *per se*, trace elements or vitamins also seem to play a largely neglected role. A high intake of fatty seafood increases circulating levels of the insulin-sensitising hormone adiponectin. As compared with a high meat intake, high intake of seafood has been reported to reduce plasma levels of the hepatic acute-phase protein C-reactive protein level in some, but not all studies. More studies are needed to confirm the dietary effects on energy intake, obesity and insulin resistance. Future studies should be designed to elucidate the potential contribution of trace elements, vitamins and undesirables present in seafood, and we argue that stratification into responders and non-responders in randomised controlled trials may improve the understanding of health effects from intake of seafood.

## Introduction

Obesity affects virtually all ages and socio-economic groups and is about to overwhelm both developed and developing countries. Excess adiposity is a well-established risk factor for overall premature mortality and major chronic diseases, including cardiometabolic diseases, type 2 diabetes (T2D), as well as cancer such as postmenopausal breast cancer and colorectal cancer^(^ ^1^ ^–^ ^3^ ^)^. Leaving genetics aside, weight gain and loss are inevitably related to energy consumed and energy used, although psychological, cultural and sociodemographic factors are all known to contribute to this energy imbalance. Besides increasing physical activity, changing dietary patterns is the single most prevailing tool to curb this escalating problem^(^ ^4^ ^)^. In this respect, the quality and type of food will also matter as certain nutrients strongly influence appetite, satiety, energy expenditure and thermogenesis, and thereby obesity development.

**Fig. 1:**
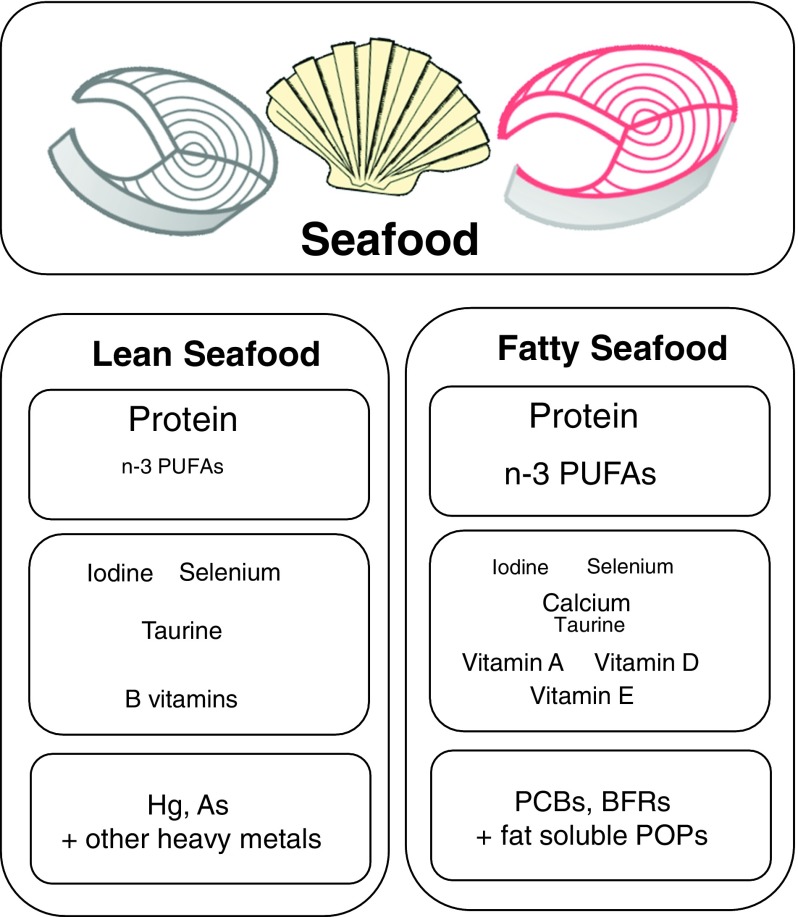
Content of nutrients and undesirables typically found in different amounts in lean and fatty seafood. Larger font size indicates higher level. PCB, polychlorinated biphenyls; BFR, brominated flame retardants; POP, persistent organic pollutants.

Lean and fatty fish are both considered nutritious and a great source of protein, iodine and various vitamins and minerals, but fatty fish contain some important nutrients in higher quantities such as *n*-3 fatty acids and vitamin D (Fig. 1). In the dietary guidelines for Americans, intake of approximately 225 g varied seafood weekly, including lean and fatty fish to provide a weekly dose of 1·75 g EPA and DHA is recommended^(^ ^5^ ^)^. According to the European Food Safety Authority (EFSA), the food-based dietary guidelines for fish consumption range from 100 to 300 g weekly in most countries^(^ ^6^ ^)^. The Nordic Nutrition Recommendations^(^ ^7^ ^)^ and the Norwegian Food-based Dietary Guidelines are somewhat higher and include 300–450 g pure fish weekly, of which 200 g should be fatty fish (salmon, trout, mackerel or herring)^(^ ^8^ ^)^.

In the USA, seafood consumption in general is reported to be as low as 63 g/week, of which 50 % is shrimp^(^ ^9^ ^)^. In contrast, in 40- to 69-year-old Japanese, a median fish consumption of 580 g weekly has been observed^(^ ^10^ ^)^. The mean intake of fish in the general Norwegian population aged 18–70 years is reported to be 450 and 300 g weekly among men and women, respectively^(^ ^11^ ^)^. In Western Norway, a median total fish intake of 530 g/week has been reported among men and women aged 46–49 and 71–74 years^(^ ^12^ ^)^ and 680 g weekly among 62-year-old patients with coronary artery disease^(^ ^13^ ^)^. Although fish intake among adults and elderly in Norway seems to live up to the guidelines, recent data indicate that the mean intake (168 g/weekly) among younger individuals (aged 9 and 13 years) does not meet the recommendations^(^ ^14^ ^)^. In addition, the frequency of fish consumption among 66 % of young European overweight adults is lower than usually recommended^(^ ^15^ ^)^. Seafood is considered an essential part of a healthy diet, but whether replacing meat with fish and seafood or increasing the intake of fish and seafood will limit the development of obesity, insulin resistance and T2D remains an open question.

Research on the health effects of fish and seafood consumption has to a large extent been focused on the content of marine *n*-3 PUFA, and a number of clinical intervention trials documenting their effects have been published. However, seafood also represents a rich source of high-quality protein and further contributes to a better nutritional status due to the content of other essential nutrients, such as vitamin D, vitamin B_12_, the B vitamins niacin and pantothenic acid, as well as the trace elements iodine and Se. On the other hand, the content of As and heavy metals such as Cd, Pb and Hg has been of concern in terms of seafood safety. Further, fatty fish in particular also contain persistent organic pollutants (POP) such as polychlorinated biphenyls, dioxins and brominated flame retardants that all have been associated with obesity and diabetes development^(^ ^16^ ^,^ ^17^ ^)^. Here, we aim to review observational studies and intervention trials related to obesity, insulin resistance and T2D with a main focus on fish or seafood consumption, but we also include studies reporting on intake of single components from seafood. Finally, we review animal trials and describe the possible mechanisms by which both fatty and lean seafood may influence the development of obesity, insulin resistance and T2D.

## Observational studies with seafood intake and obesity

Individuals adhering to the so-called prudent diet, characterised by a higher consumption of non-hydrogenated fat, vegetables, eggs, fish and other seafood, are less likely to be obese than individuals having a high intake of refined grains, red meats, processed meats, French fries, condiments and regular sugar-containing soft drinks^(^ ^18^ ^)^. Healthy dietary patterns comprising intake of seafood have also been associated with a low BMI in Japan^(^ ^19^ ^)^ and Jakarta^(^ ^20^ ^)^. Additionally, a few prospective studies have investigated the relationship between fish consumption and body-weight gain. In the European Prospective Investigation into Cancer and Nutrition (EPIC) study, comprising 249 558 women and 95 199 men from ten European countries, overall fish consumption was weakly positively associated with increase in body weight in women, but not in men^(^ ^21^ ^)^. It has to be mentioned that in the EPIC study the median follow-up period was only 5 years, and among women, data differed between the different countries, i.e. in Greece, the Netherlands and UK negative associations for total fish intake and body-weight gain were observed^(^ ^21^ ^)^. It is not yet known if the differences relate to cultural differences regarding how seafood-containing meals are composed, use of condiments, or preparation methods. In a Norwegian study using data from two cross-sectional surveys, the population-based Tromsø 4 and Tromsø 6 studies (http://tromsoundersokelsen.no), data from 4528 individuals with a follow-up of 13 years showed that individuals with an intake of fatty fish once/week or more exhibited increased waist circumference compared with those eating fatty fish less than once/week^(^ ^22^ ^)^. In contrast, men who consumed lean fish more than once/week had decreased waist circumference, but this association was not statistically significant after multiple adjustments^(^ ^22^ ^)^. However, data from this study indicate that the type of fish also may be of importance when evaluating intake of seafood and obesity development. In a large American study, the association between 4-year changes in consumption of different protein sources and body weight has been investigated in three prospective US cohorts over a 16- to 24-year follow-up period (Nurses’ Health Study, Nurses’ Health Study II, and Health Professionals Follow-Up Study) including 120 784 men and women without chronic disease or obesity at baseline^(^ ^23^ ^)^. Whereas increased intake of protein from meats, chicken with skin and regular cheese was associated with weight gain, increased intake of seafood together with peanut butter, walnuts, other nuts, chicken without skin, yogurt and low-fat cheese was associated with weight reduction^(^ ^23^ ^)^. However, it should be mentioned that changes in most protein foods were inversely correlated with changes in carbohydrate at baseline. The authors emphasise that dietary replacements, especially replacing protein-rich food for carbohydrate-rich foods, are crucial for long-term weight maintenance. Originally, Iso *et al.* ^(^ ^24^ ^)^ reported that participants in the Nurses’ Health Study with a high intake of fish had a higher risk of obesity. However, these women also had a high intake of poultry, which is rich in the *n*-6 PUFA linoleic acid^(^ ^25^ ^)^. This may be of importance as intake of poultry and linoleic acid has been positively correlated with obesity^(^ ^26^ ^)^. Further, the possible protective effect of marine *n*-3 PUFA on obesity development^(^ ^27^ ^–^ ^29^ ^)^ may be counteracted by linoleic acid^(^ ^26^ ^)^.

A great number of publications have described the health-beneficial effects of marine *n*-3 PUFA on obesity-related disorders. Hence, fatty fish such as salmon, herring and mackerel have been considered health beneficial largely due to their high content of marine *n*-3 PUFA. As a high consumption of meat is associated with weight gain and consumption of fish and seafood with weight loss^(^ ^23^ ^)^, exchanging meat for seafood should, in theory, be beneficial in terms of weight loss. However, only a limited number of human intervention studies using fatty fish have actually been performed on obese subjects.

## Intervention studies with fatty seafood and obesity

Results from published intervention studies suggest that seafood may accelerate weight loss induced by energy restriction (Table 1). In a study by Mori *et al.* ^(^ ^30^ ^)^, sixty-three overweight patients that underwent treatment for hypertension were randomised to a daily fish meal, a weight-loss regimen, the two regimens combined, or a control group for 16 weeks. The fish meals comprised Greenland turbot, canned sardines, canned tuna or canned salmon, providing an average of 3·65 g marine *n*-3 PUFA/d. The subjects assigned to the weight-loss groups had a dietary programme in which their daily energy intake was individually decreased by 2000–6500 kJ/d for 12 weeks to achieve a weight loss of 5–8 kg. There was no significant change in body weight in the seafood and no-seafood groups that maintained their usual energy intake, but increased weight loss was observed when energy restriction was combined with a daily fatty fish meal. A strength of this trial is the measurement of *n*-3 and *n*-6 PUFA in plasma indicating compliance with fish intake in the fish groups.

**Table 1:** Randomised controlled trials (RCT) with fatty and/or lean fish on obesity

Author	Subjects	Health	Design	Background diet	Intervention groups	Duration	Main results
Mori *et al.* (1999)^(^ ^30^ ^)^	*n* 63 42 M 21 F Mean age: 54·1 (sem 1·8) years Age range: 40–70 years	Hypertension BMI >25 kg/m^2^ Mean BMI: 34·9 (sem 1·1) kg/m^2^	RCT	Self-selected	(1) Control (2) Fish (3) Weight loss (energy-restricted diet) (4) Fish + weight loss Fish groups: including 3·65 g/d *n*-3 PUFA (turbot, sardines, tuna, salmon)	16 weeks	Weight decreased by 5·6 (sem 0·8) kg in energy restriction groups. NS differences in weight loss in seafood and no-seafood groups maintaining usual energy intake
Thorsdottir *et al.* (2007)^(^ ^31^ ^)^; Ramel *et al.* (2010)^(^ ^32^ ^)^	*n* 278 120 M 158 F Mean age: 38·9 (sd 5·4) years Age range: 20–40 years	Healthy Mean BMI: 30·1 (sd 1·4) kg/m^2^ BMI range: 27·5–32·5 kg/m^2^	RCT	Self-selected	Energy-restricted diets (1) Control (sunflower capsules, no seafood) (2) Lean fish (3 × 150 g cod/week) (3) Fatty fish (3 × 150 g salmon/week)=2·1 g/d *n*-3 PUFA (4) Fish oil (capsules, no seafood)=1·3 g/d *n*-3 PUFA	8 weeks	Weight and waist circumference decreased significant more in fatty fish (–7·0±3·5 kg), lean fish (–6·6±2·8 kg) and fish oil groups (–6·7±3·6 kg) (energy-restricted diets) compared with control –5·3±3·0 kg) in male subjects
Ramel *et al.* (2009)^(^ ^74^ ^)^	*n* 126 Age range: 20–40 years	Healthy Mean BMI: 30·2 (sd 1·4) kg/m^2^27·5–32·5 kg/m^2^	RCT	Self-selected	Energy-restricted diets (1) Control (no seafood) (2) Lean fish (3 × 150 g cod/week) (3) Lean fish (5 × 150 g cod/week)	8 weeks	Dose–response relationship; weight loss increased significantly with increasing doses of cod: cod 3 ×/week –0·67 kg; cod 5 ×/week –1·73 kg compared with control
Aadland *et al.* (2016)^(^ ^76^ ^)^	*n* 20 7 M 13 F Mean age: 50·6 (sem 3·4) years	Healthy Mean BMI: 25·6 (sem 0·7) kg/m^2^	RCT, cross-over	3 weeks run-in period with diet in accordance with Norwegian recommendations	No energy-restriction (1) Lean seafood 7 d/week (2) Non-seafood 7 d/week Fish: cod, pollock, saithe, scallops	2 × 4 weeks, 5 weeks washout	No diet effect on body composition

M, male; F, female.

In line with this, Thorsdottir *et al.* ^(^ ^31^ ^)^ and Ramel *et al.* ^(^ ^32^ ^)^ have demonstrated that inclusion of fatty fish, or fish oil as part of an energy-restricted diet, significantly increased weight loss in young overweight adults. In this study, 278 overweight men and women (20–40 years) from Iceland, Spain and Ireland were subjected to weight loss induced by 30 % energy restriction for 8 weeks. One group received 1·3 g of marine *n*-3 PUFA from capsules/d and one group received three portions of 150 g salmon/week, corresponding to an average daily intake of 2·1 g marine *n*-3 PUFA during the 8 weeks of energy restriction. The diets did not vary in their influence on weight loss in women, but in men inclusion of either fatty fish or fish oil in the diet with energy restriction resulted in approximately 1 kg greater weight loss after the first 4 weeks compared with a similar diet without seafood or *n*-3 PUFA supplement.

In line with the possible ability of marine *n*-3 PUFA to accentuate weight loss induced by energy restriction, Kunešová *et al.* ^(^ ^33^ ^)^ have demonstrated greater weight loss in severely obese women when 2·8 g marine *n*-3 PUFA/d were included in an energy-restricted diet during a 21 d trial. Of note, a combined intervention using marine *n*-3 PUFA and minor energy restriction exerted synergism in the prevention of obesity also in mice^(^ ^34^ ^)^. Further, Kabir *et al.* ^(^ ^35^ ^)^ reported that 3 g fish oil/d reduced total fat mass and adipocyte size in a 2-month randomised controlled trial (RCT) with type 2 diabetic women. Good compliance was seen in all the above-mentioned trials. In line with Kabir *et al.* ^(^ ^35^ ^)^, an inverse association has been observed in patients between abdominal obesity and amount of marine *n*-3 PUFA in adipose tissue samples^(^ ^36^ ^)^ and also between the amount of marine *n*-3 PUFA in subcutaneous adipose tissue and reduced adipocyte size^(^ ^37^ ^)^. However, other similar trials have failed, and a lack of consensus between animal trials and human intervention studies apparently exists^(^ ^38^ ^)^. A meta-analysis of the potential of *n*-3 PUFA to reduce obesity in humans with a description of the lack of consistency in study designs was recently published elsewhere^(^ ^39^ ^)^, and will not be further discussed here. It should, however, be mentioned that a small reduction in body fat mass is not always accompanied by reduced body weight. For instance, in a cross-over trial, Couet *et al.* ^(^ ^40^ ^)^ reported that replacement of 6 g of dietary fat (butter, olive oil, sunflower-seed oil and peanut oil) with 6 g of marine *n*-3 PUFA/d given as capsules for 3 weeks led to a reduced body fat mass without a concomitant reduction in body mass. Still, a meta-analysis^(^ ^41^ ^)^ where twelve trials met the eligibility criteria reported on a significantly higher weight loss in the intervention groups (fatty fish or marine *n*-3 PUFA) compared with the control groups.

Fatty fish is a rich dietary source of fat-soluble vitamins, including vitamin D. Obesity often coexists with low intake of Ca and with vitamin D insufficiency^(^ ^42^ ^)^. Dietary Ca may lead to a negative energy balance by its ability to reduce intestinal fat absorption because of formation of insoluble Ca–fatty acid soaps, which pass unabsorbed through the intestinal tract and are excreted in the faeces. A number of meta-analyses have investigated whether a sufficient Ca intake may prevent or reduce obesity, but there is a lack of consensus^(^ ^43^ ^)^. The link between vitamin D and obesity is not yet completely understood, but obesity-related vitamin D deficiency has been related to reduced bioavailability of vitamin D from cutaneous and dietary sources because of its deposition in body fat compartments^(^ ^44^ ^)^. A very limited number of studies examining the effect of vitamin D supplementation on weight loss have been performed, and two recent reviews on the topic concluded that although epidemiological associations are clear, more intervention studies are needed to conclude on whether increasing vitamin D intake can attenuate weight gain or augment weight loss^(^ ^42^ ^,^ ^45^ ^)^. Thus, whether improved vitamin D and/or Ca status by fatty fish intake could contribute to reduced obesity needs to be further elucidated.

## Animal trials with fatty seafood and potential mechanisms of actions

In view of the promising rodent studies performed by us and others documenting the ability of marine *n*-3 PUFA to attenuate and/or totally prevent high-fat diet-induced obesity in rodents^(^ ^26^ ^,^ ^34^ ^,^ ^46^ ^–^ ^56^ ^)^, one would expect fatty fish to effectively attenuate obesity. However, only a limited number of studies have been published, and the results in terms of the potential anti-obesogenic effect from experiments using fatty fish are far less convincing. Still, several reports from Sweden suggest that herring may have some anti-obesogenic properties, including an experiment where high-fat/high-sucrose diets supplemented with either minced herring fillets or minced beef were fed to male LDL receptor-deficient mice for 16 weeks. Despite increased body weight, body composition was equal and the size of adipocytes in epididymal fat was reduced in herring-fed mice compared with beef-fed mice^(^ ^57^ ^)^. Further, it was demonstrated that offspring of herring-fed C57BL/6 mice were less obese than offspring of beef-fed dams at 9 week of age. The fatty acid composition in the breast milk was strongly affected by inclusion of herring in the maternal diet, and this translated into increased levels of *n*-3 PUFA in several tissues of the offspring of dams fed the herring-containing diet^(^ ^58^ ^)^. Further, rats fed high-energy diets with herring exhibited smaller adipocytes in the mesenteric adipose tissue depots than rats fed high-energy diets with chicken^(^ ^59^ ^)^. Conversely, mice fed very high-fat diets with salmon became more obese than mice fed the ‘control’ casein-based diets with similar macronutrient composition^(^ ^60^ ^)^. However, although casein is commonly used as the protein source in commercially available rodent diets, casein may not represent an adequate reference control compared with many other protein sources as casein has anti-obesogenic properties in obesity-prone C57BL/6J mice^(^ ^61^ ^,^ ^62^ ^)^.

Interestingly, the fatty acid composition in salmon feed and, hence, salmon fillets may be of importance^(^ ^63^ ^,^ ^64^ ^)^. Feeding salmon aquatic feed with 50 % replacement of the traditionally used marine oils with vegetable oils, soyabean oil in particular, resulted in a profoundly increased *n*-6:*n*-3 PUFA ratio in salmon fillets^(^ ^65^ ^)^. Fatty acid composition in tissues and erythrocytes in mice fed diets containing the salmon mirrored the fatty acid composition of the fillets, and an increased *n*-6:*n*-3 PUFA ratio was associated with a more obese phenotype^(^ ^63^ ^,^ ^66^ ^)^. Conversely, an increased ratio of *n*-3:*n*-6 PUFA in the fish feed, salmon fillets, and in erythrocytes collected from the mice fed the salmon was accompanied with reduced adipose tissue mass and reduced abundance of arachidonic acid (AA) in the phospholipid pool in the livers of the mice^(^ ^63^ ^,^ ^64^ ^,^ ^66^ ^)^. The levels of hepatic ceramides and AA-derived pro-inflammatory mediators decreased, whereas the abundance of oxylipins derived from EPA and DHA was increased^(^ ^66^ ^)^. Similarly, in plasma and liver, the levels of AA-derived endocannabinoids, 2-arachidonoylglycerol and anandamide, N-arachidonoylethanolamine, decreased, whereas the levels of EPA- and DHA-derived endocannabinoids increased^(^ ^63^ ^,^ ^66^ ^)^. It is well known that endogenously produced AA-derived endocannabinoids can promote obesity^(^ ^26^ ^,^ ^67^ ^)^. Hence, reduced production of AA-derived and/or increased production of *n*-3-derived endocannabinoids and oxylipins may explain why the *n*-6:*n*-3 PUFA ratio in salmon modulates metabolism in mice consuming the salmon.

Dietary composition plays an important role in shaping the microbiota, and it is currently widely accepted that the composition of the gut microbiota is linked to obesity^(^ ^68^ ^)^. Compared with diets rich in SFA, a diet rich in marine *n*-3 PUFA led to a higher Bacteroidetes:Firmicutes ratio after 14 weeks^(^ ^69^ ^)^. Although challenged, a decreased Bacteroidetes:Firmicutes ratio has traditionally been associated with obesity^(^ ^70^ ^)^. Further, it is reported that mice fed fish oil have increased levels of *Akkermansia muciniphila* ^(^ ^71^ ^)^, which has been associated with protection against diet-induced obesity^(^ ^72^ ^)^. Of interest, it was recently demonstrated that a specific protein isolated from the outer membrane of *A. muciniphila*, named Amuc_1100, is able to improve the gut barrier and partly recapitulates the beneficial effects of *A. muciniphila* ^(^ ^73^ ^)^. However, to what extent fatty seafood is able to modulate the composition and function of the gut microbiota warrants further investigation.

## Intervention studies with lean seafood and obesity

Components in fish besides the marine *n*-3 PUFA are often overlooked, but in the context of weight management, several human intervention studies suggest that components of lean seafood also may be of importance (Table 1). First, in the previously mentioned study by Thorsdottir *et al.* ^(^ ^31^ ^)^, it was demonstrated that inclusion of lean fish, 150 g cod for 3 d per week, in an energy-restricted diet was as efficient as salmon to increase weight loss by approximately 1 kg in overweight young males. Increasing the fish intake to 150 g cod for 5 d per week resulted in a 1·7 kg significantly greater weight loss than intake of an isoenergetic diet^(^ ^74^ ^)^. Second, in a recent 8-week intervention study with free-living subjects, it was shown that daily self-administration of capsules with 3 g of fish protein per d for 4 weeks decreased the percentage of body fat and increased the percentage of muscle in overweight adults^(^ ^75^ ^)^. However, during the last 4 weeks of the study, when the daily protein supplementation was increased to 6 g/d, the differences in body composition disappeared^(^ ^75^ ^)^. In a cross-over study with two 4-week diet periods in which the participants were given daily lunch and dinner meals with either lean seafood or non-seafood (mainly lean meat), we did not observe differences in body composition between diets in healthy adults. Of importance, energy intake was kept equal for each individual between lean seafood and the non-seafood diet periods^(^ ^76^ ^)^. Despite no differences in body composition, 4 weeks of high lean seafood as compared with no seafood intake altered lipid and glucose metabolism, as evident from changes in fasting and postprandial serum metabolites^(^ ^76^ ^,^ ^77^ ^)^ as well as differences in the urine metabolome^(^ ^78^ ^)^. As obesity development may take years, it is possible that the above-mentioned studies were of too short duration to detect any sustained difference in body composition, but may indicate prevention against obesity. Still, inclusion of seafood in an energy-restricted diet may be useful to increase weight loss. However, presently there is not sufficient evidence from RCT to state that seafood affects body composition differently from other protein-rich foods when individuals are consuming their habitual amount of energy.

Although still controversial, different types of high-protein diets are popular. Given the high protein content and virtually no carbohydrate content in lean fish, an increased intake of fish would necessarily lead to increased protein intake. Increasing dietary proteins increase satiety and diet-induced thermogenesis, and during weight loss dietary proteins have a favourable effect on body composition due to sparing of fat-free mass^(^ ^79^ ^–^ ^82^ ^)^. Moreover, in a European multicentre trial, it was demonstrated that just a modest increase in dietary protein intake effectively prevented weight regain after a major weight loss in obese subjects^(^ ^83^ ^,^ ^84^ ^)^. In contrast to anecdotal suggestions, seafood proteins have been demonstrated to be more filling than proteins from red meat and chicken^(^ ^85^ ^,^ ^86^ ^)^.

Uhe *et al.* ^(^ ^86^ ^)^ compared the acute satiating effect of beef, chicken fillet without skin and gummy shark meals by administrating grilled whole chunks of 50 g of protein of each type together with 200 ml of water to the subjects participating the study. The meal sizes were not reported, but as lean seafood contains more water than terrestrial meats, it is likely that the gummy shark meal was larger than the two other meals. The subjects rated repeatedly how hungry or full they felt during 180 min following commencement of the meals. Satiety was greater after the seafood meal than after intake of meals based on the other protein sources and this was related to lower digestion rate and a higher postprandial tryptophan:large neutral amino acid ratio. The authors hence suggested involvement of the neurotransmitter serotonin (5-hydroxytryptamine) as one of the signals mediating the satiety. A higher postprandial tryptophan:large neutral amino acid ratio would imply that more tryptophan enters the brain. As a result, serotonin synthesis would increase and possibly interact within the hypothalamus with endogenous orexigenic (neuropeptide Y/Agouti-related protein) and anorectic (α-melanocyte stimulating hormone) peptides^(^ ^86^ ^)^.

Borzoei *et al.* ^(^ ^85^ ^)^ served healthy males an isoenergetic protein-rich (47 energy percent (%E) protein) lunch meal, consisting of a dish containing either minced cod or minced beef. An *ad libitum* standardised evening meal was served 4 h after the start of the lunch meals. Food intake was measured, and appetite was rated by visual analogue scales. The results showed that the point estimates were somewhat lower for hunger and higher for satiety, but no significant differences were observed. However, in participants who ate the fish lunch meal, energy intake at the evening meal was significantly lower and the subjects did not feel less satiated, and no subsequent energy compensation after the evening meal was found on the test day^(^ ^85^ ^)^. In contrast to the results from the study of Borzoei *et al.* ^(^ ^85^ ^)^, we found no difference on appetite sensation or energy intake after consumption of balanced meals (26 %E protein) with either cod or lean veal in a recent study^(^ ^87^ ^)^. Moreover, we observed no differences in plasma levels of ghrelin, a known orexigenic hormone.

Five intervention studies have been performed with lean seafood as part of a lean white meat diet in comparison with a lean red meat diet. The primary endpoint of these studies was plasma lipids, but they also recorded energy intake. In a cross-over study with 129 healthy American females (*n* 55) and males (*n* 74) aged 23–70 years, the participants consumed at least 140 g/d of either lean beef, or poultry (chicken and turkey) 4 d/week and fish (cod, perch and sole) 3 d/week for two diet periods of 3 months each^(^ ^88^ ^)^. Even though the difference was not significant, the mean energy intake was 9 % lower for both sexes in the lean white meat diet period (including lean fish) relative to the energy intake in the lean red meat diet period. In another cross-over study from the same group using similar conditions, energy intake during the lean white meat diet period was lower (–9 % in females and –16 % in males) as compared with energy intake during the lean red meat diet period, but did not reach statistical significance^(^ ^89^ ^)^. A cross-over study in 145 hypercholesterolaemic American men and women (18–75 years) compared the effect of consuming at least 170 g/d for 5–7 d/week of lean red meat (beef, veal or pork) with the same amount of lean white meat (poultry or fish) for two diet periods of 9 months^(^ ^90^ ^)^. Energy intake was significantly lower (–4·5 %; *P*=0·004) during the lean white meat diet period as compared with energy intake during the lean red meat period. Neither data on body weight nor on the type or amount of lean fish consumed were specified in this study^(^ ^90^ ^)^. Data from diet period 1 in the cross-over study by Hunninghake *et al.* ^(^ ^90^ ^)^ were published separately as a parallel-arm study with eighty-nine subjects in the lean red meat group, and 102 subjects in the lean white meat group. In diet period 1, energy intake tended (*P*=0·06) to be reduced in the lean white meat group relative to the lean red meat group. Concomitantly, changes in body weight during the 9 months’ study were +0·8 kg for the lean red meat group and –0·5 kg in the lean white meat group, but the difference was not significant^(^ ^91^ ^)^. Finally, in a cross-over study, thirty-nine hypercholesterolaemic South-African participants, aged 20–53 years, consumed prudent diets with either lean beef (5 d/week) and lean mutton (2 d/week) or with skinless chicken (5 d/week), hake (1 d/week) and pilchards or tuna (1 d/week) for two diet periods of 6 weeks^(^ ^92^ ^)^. Both prudent diets reduced energy intake as compared with baseline intake, but the lean white meat diet reduced energy intake more than the lean red meat diet. The changes in body weight were –0·5 kg for the red meat diet period, and –1·2 kg for the lean white meat diet period, but the difference was not significant^(^ ^92^ ^)^. Taken together, the inclusion of lean seafood, in particular at the expense of red meat, is likely to reduce energy intake and, hence, body-weight gain. Unfortunately, however, these studies have to our knowledge not reported on hormone levels related to satiety.

The underlying mechanism governing the possible preventive effect of lean seafood on body-weight gain is not clear. However, one possible mechanism is the generation of bioactive peptides through the digestion of food proteins. Bioactive peptides tend to have two to twenty amino acid residues, and may either be effective after absorption in the gut or they may induce a local effect in the gastrointestinal tract^(^ ^93^ ^)^. These bioactive peptides have been suggested to influence energy intake and body-weight regulation^(^ ^94^ ^)^. In addition, lean seafood is generally a rich source of iodine^(^ ^95^ ^)^, which may be of relevance as inadequate iodine status is a major threat worldwide, and approximately two billion individuals are estimated to have inadequate iodine intake^(^ ^96^ ^)^. Little is known about the relationship between BMI and iodine status, but obesity was recently associated with a higher risk of iodine deficiency, which might lead to hypothyroidism^(^ ^97^ ^)^. Still, whether iodine present in fish and seafood could play a role in the prevention of obesity remains an open question.

## Animal trials with lean seafood, obesity and potential mechanisms of actions

Animal studies suggest that lean seafood is less obesogenic than meat from terrestrial animals. Rats fed a high-fat diet containing Alaska pollock as the protein source gained less visceral fat than rats fed chicken^(^ ^98^ ^)^. Further, we have observed lower adiposity in mice fed a Western diet containing a mixture of lean seafood (ling, rosefish, cod, wolf fish) and muscle from Canadian scallop than in mice fed a Western diet containing a mixture of skinless chicken breast, pork tenderloin and beef sirloin^(^ ^99^ ^)^. This was accompanied with reduced energy intake (8 % lower in seafood-fed mice), but we also observed lower feed efficiency and a higher spontaneous locomotor activity. In a comparable dietary setting, obesity development was reduced by exchanging meat from lean pork with cod^(^ ^100^ ^)^. Here, we included a second group of pork-fed mice that were pair-fed with the group of mice fed cod. The pair-fed mice were mildly energy restricted, as the *ad libitum* cod-fed mice consumed 6 % less energy than *ad libitum* pork-fed mice. Still, feed efficiency in the pair-fed mice consuming the pork-based feed was significantly higher than that of cod-fed mice. Whereas adiposity in the cod-fed mice was significantly lower than in *ad libitum* pork-fed mice, adiposity in the pair-fed pork group was in between. Fat mass in the pair-fed mice was not significantly different from either of the *ad libitum*-fed groups. Feed efficiency and adipose tissue mass were also lower in mice fed high-fat diets (67 %E fat, 18 %E sucrose and %E protein) with a mixture of cod and scallop than in mice fed the high-fat diet based on skinless chicken fillet^(^ ^62^ ^)^. Further, spontaneous locomotor activity tended to be decreased in chicken-fed mice when shifting from low-fat to high-fat diets. Together, pair-feeding experiments suggest an important contribution of higher spontaneous locomotor activity and decreased feed efficiency to the anti-obesogenic effect, but decreased energy intake also appears to contribute when animals are fed *ad libitum*. Of note, whereas no difference was observed in first-choice preference between the diets containing lean seafood and lean meat, mice were observed to eat significantly more meat-containing diets than seafood-containing diets during the following 6 h^(^ ^99^ ^)^. Although several studies have reported reduced energy intake when mice are fed diets containing lean seafood compared with lean meat, the underlying mechanisms by which seafood may increase satiety have not yet been elucidated.

The anti-obesogenic effect of lean seafood may be related to the content of taurine and glycine. We have demonstrated that intake of taurine and glycine was negatively correlated with adiposity in mice fed either chicken, cod, crab or scallop in high-fat, high-sucrose diets^(^ ^101^ ^)^. This is in line with experiments reporting that both taurine^(^ ^102^ ^,^ ^103^ ^)^ and glycine^(^ ^104^ ^,^ ^105^ ^)^ can reduce fat mass in rodents. Further, intake of diets containing a fish protein hydrolysate, rich in taurine and glycine, reduced adipose tissue mass in rats^(^ ^106^ ^,^ ^107^ ^)^. In the rat experiments, the reduced adiposity was accompanied with elevated plasma bile acid concentration^(^ ^108^ ^)^. Bile acid-mediated activation of farnesoid X receptor and TGR5 (bile acid membrane receptor) may affect metabolism and energy expenditure in rats. However, we did not observe differences in circulating bile acids in mice, despite large differences in intake of glycine and taurine^(^ ^101^ ^)^. It was recently demonstrated that taurine supplementation was able to prevent high-fat diet-induced weight gain and increased visceral fat mass^(^ ^102^ ^)^. Further, taurine supplementation alleviated high-fat diet-induced disturbances in circadian rhythms, such as 24 h patterns of plasma insulin and leptin, possibly by normalisation of high-fat diet-induced down-regulation expression of clock genes in pancreatic islets^(^ ^102^ ^)^. We have observed that cod/scallop-fed mice tended to be more active than casein- and chicken-fed mice in the dark phases^(^ ^101^ ^)^. Thus, it is possible that seafood may attenuate high-fat diet-induced disturbances in the circadian rhythm. However, further experiments are needed to identify the mechanisms behind the observed differences.

**Fig. 2:**
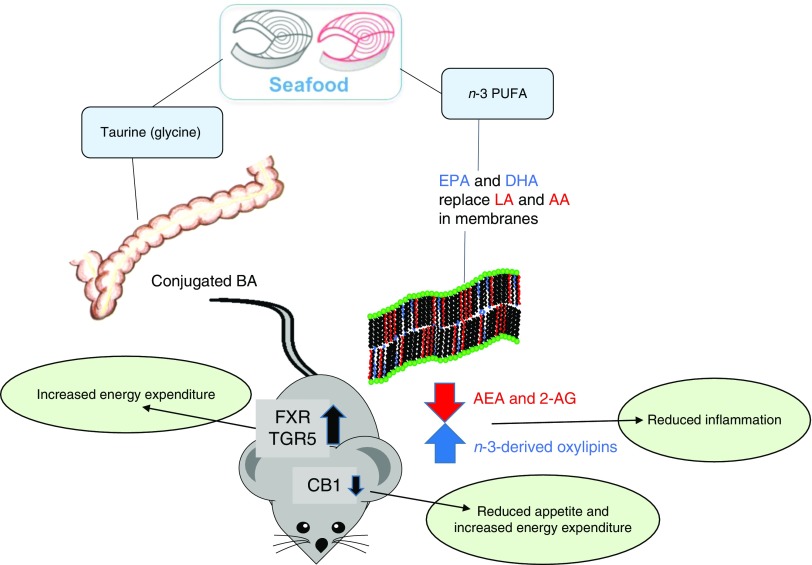
Potential mechanisms by which seafood intake may influence energy intake and metabolism based on data from animal studies. BA, bile acids; LA, linoleic acid; AA, arachidonic acid; AEA, N-arachidonoylethanolamine; 2-AG, 2-arachidonoylglycerol; FXR, farnesoid X receptor; TGR5, bile acid membrane receptor (also known as Gpbar1); CB1, cannabinoid receptor type 1.

Compared with fatty seafood, the amount of marine *n*-3 PUFA present in lean seafood is low. However, it is important to note that a large fraction of the phospholipids present in lean seafood contains EPA and DHA^(^ ^109^ ^,^ ^110^ ^)^. It has been reported that the bioavailability of EPA and DHA as well as their ability to modulate endocannabinoid signalling and the anti-obesogenic effect are higher when they are present in phospholipids than in TAG^(^ ^111^ ^,^ ^112^ ^)^. However, we recently demonstrated that addition of phospholipid-bound, but not TAG-bound, *n*-3 PUFA to a pork-based diet led to a small increase in weight gain^(^ ^113^ ^)^. Further, freezing initiates hydrolysis of the phospholipids present in the fillet, but the anti-obesogenic effect of frozen stored cod was more pronounced than fresh cod^(^ ^113^ ^)^. Still, feeding mice Western diets where meat from lean pork was exchanged with stored frozen cod for 12 weeks lowered the *n*-6:*n*-3 ratio in liver phospholipids and in erythrocytes^(^ ^100^ ^)^. Concomitantly, lower circulating levels of N-arachidonoylethanolamine and 2-arachidonoylglycerol, the two major AA-derived endocannabinoids, were observed. The accompanied reduced adiposity in cod-fed mice suggested that the content of marine *n*-3 PUFA is sufficient to modulate endocannabinoid signalling and obesity development in mice. The endocannabinoid receptor CB_I_ is an important regulator of appetite, and although not directly shown, a reduced ratio of *n*-6:*n*-3-derived endocannabinoids may also reduce appetite. The endocannabinoid receptor CB_I_ is suggested to influence gut permeability via interaction with the gut microbiota, and may thus link the gut microbiota to adiposity^(^ ^114^ ^)^. Comparison of the gut microbiomes of mice fed Western diets with lean seafood or meat from lean terrestrial animals revealed significant differences in the relative abundance of operational taxonomic units belonging to the orders *Bacteroidales* and *Clostridiales* ^(^ ^99^ ^)^. Based on functional analyses, it appeared that the gut microbiota in seafood-fed mice had higher capacity for amino acid transport and biosynthesis of tyrosine and phenylalanine. The gut microbiota in meat-fed mice appeared to have higher capacity for lysine degradation and had higher abundance of genes involved in the pentose phosphate and glucoronate pathways. Further, intake of taurine has been demonstrated to reduce the abundance of Proteobacteria, especially *Helicobacter* and increase SCFA content in faeces^(^ ^115^ ^)^. Intake of non-digestible carbohydrates may lead to production of SCFA, mainly acetate, propionate and butyrate, that may enter the systemic circulation and counteract obesity in both rodents and humans^(^ ^116^ ^)^. However, the importance of the gut microbiota in mediating the anti-obesogenic effect of lean seafood in animal studies is not yet known. Based on findings from animal studies, potential mechanisms linking intake of lean seafood to effects on energy intake and metabolism are presented in Fig. 2.

## Observational data with seafood intake and development of type 2 diabetes

As mentioned earlier, the large prospective cohort study by Smith *et al.* ^(^ ^23^ ^)^ (>120 000 participants) showed that over time higher intake of seafood, chicken fillet without skin and plain- or artificially sweetened yoghurt was associated with reduced body weight^(^ ^23^ ^)^. It is generally accepted that obesity is positively associated with the development of insulin resistance^(^ ^117^ ^,^ ^118^ ^)^, which may progress to T2D. In obese subjects who develop insulin resistance and T2D, adipose tissue dysfunction may be one underlying mechanism^(^ ^119^ ^)^. Thus, if seafood intake can prevent obesity, there is also a rationale for seafood intake to prevent insulin resistance, yet the potential impact of seafood consumption on the development of insulin resistance and T2D is not fully clarified.

In prospective cohort studies the results are inconclusive as fish intake in general has been associated with reduced fasting plasma glucose in participants from Cyprus^(^ ^120^ ^)^, with lower risk of T2D in Japanese men, but not women^(^ ^121^ ^)^, and with lower incidence of T2D in Chinese women^(^ ^122^ ^)^. Intake of lean fish, but not fatty fish, was found to have beneficial effects on metabolic syndrome components^(^ ^22^ ^)^ and T2D^(^ ^123^ ^)^ in Norwegians. Non-fried fish consumption was associated with lower incidence of the metabolic syndrome in American adults^(^ ^124^ ^)^. Intake of total, lean and fatty fish was found to be beneficial for reducing the risk of T2D, whereas shellfish increased the risk in participants from England^(^ ^125^ ^)^. Intake of shellfish and fried fish was also associated with increased risk of T2D in men from Sweden^(^ ^126^ ^)^. Other results from prospective cohort studies indicate that higher seafood intake in general did not prevent the development of T2D^(^ ^126^ ^)^, or was even associated with moderately increased incidence of T2D^(^ ^127^ ^–^ ^129^ ^)^.

Some of the discrepancy in the varying outcomes from the different prospective cohort studies might be related to geographical differences since meta-analysis of prospective studies performed in Western countries found positive associations (USA) or no associations (Europe), whereas analysis of studies performed in Eastern countries (Asia and Australia) found inverse associations between seafood consumption and risk of T2D development^(^ ^130^ ^–^ ^132^ ^)^. However, it is also likely that some of the discrepancy in the different prospective cohort studies is caused by the use of semi-quantitative FFQ that may cause erroneous food intake reporting. Further, differences in intake of fish *v.* other protein sources related to cultural dietary habits and meal compositions, use of condiments as well as total energy intake probably differ between Western and Eastern populations. Additionally, an ecological study including forty-one countries with different sociodemographic characteristics reported between diabetes, obesity and total fish and seafood consumption showing that the prevalence of T2D increased significantly with obesity in countries with low seafood consumption, and further that a high intake of fish and seafood was associated with reduced risk for diabetes in countries with a high prevalence of obesity^(^ ^133^ ^)^. It is also possible that preparation methods of the fish or dietary contaminants in fish may influence the relationship^(^ ^126^ ^,^ ^134^ ^)^. It should, however, be mentioned that an unbiased assessment of dietary intake has been used in some recent studies by analysing multiple biomarkers in blood and associated the levels of these biomarkers with glucose tolerance status and, furthermore, used measurements of such biomarkers for the prediction of T2D. For example, in the study by Savolainen *et al.* ^(^ ^135^ ^)^, use of multiple biomarkers indicated that a higher consumption of fatty fish, whole grains and vegetable oils was associated with better glucose tolerance and reduced risk of T2D.

## Acute effects of fatty fish intake on postprandial glucose metabolism

The development of insulin resistance and T2D often takes several years. It is therefore very difficult, not to say impossible, to perform RCT to study how specific dietary patterns influence the development of these metabolic disorders. Rather it is common practice to study the impact of diets on markers of glucose metabolism and insulin sensitivity such as serum concentrations of glucose or insulin. One cross-over study with healthy, overweight Swedish men (aged 41–67 years; *n* 17) compared the acute effect of ingesting 150 g of pickled or baked herring with 150 g of baked beef in complex test meals^(^ ^136^ ^)^. The test meals were balanced in relation to fat and protein intake, but not to carbohydrates in the case of pickled herring. Compliance regarding PUFA changes in plasma was good. The postprandial insulin response was equal after intake of baked herring and baked beef, but higher after the pickled herring meal, likely reflecting the higher carbohydrate content in this meal (81 *v.* 47 g in the two other meals)^(^ ^136^ ^)^.

## Frequent high intake of fatty fish and effects on insulin sensitivity

Several RCT have tested the effect of a frequent intake of seafood on markers of glucose regulation and insulin sensitivity (Table 2). Intake of one daily fatty fish meal in combination with light or moderate exercise was studied in Australian overweight T2D subjects (aged 30–65 years) who were not taking insulin^(^ ^137^ ^)^. The daily fish intake varied depending on the endogenous fat content of the chosen fish species (Greenland turbot/halibut about 200 g/d, canned salmon about 54 g/d, canned tuna about 102 g/d and canned sardines about 106 g/d), and was dosed to provide 3·65 g *n*-3/d. After 8 weeks’ intervention, individuals with an intake of one daily meal with fatty fish exhibited significantly elevated levels of glycated Hb (HbA1c) and self-reported blood glucose, but moderate exercise in combination with fatty fish improved glycaemic control more than exercise alone did^(^ ^137^ ^)^. In a later study from the same group, the effect of daily intake of the same type and amounts of fatty fish was studied for 16 weeks in Australian overweight patients that suffered from and were medicated for hypertension^(^ ^30^ ^)^. Consumption of fatty fish was examined independently and in combination with weight loss. Even though the differences did not reach statistical significance, daily intake of one meal with fatty fish for 16 weeks slightly elevated fasting blood glucose and insulin concentrations, as well as AUC following a 75 g oral glucose tolerance test as compared with the control group. In contrast, the combination of daily fatty fish intake and weight loss was more efficient in improving glucose metabolism than weight loss alone^(^ ^30^ ^)^.

**Table 2:** Randomised controlled trials (RCT) with fatty and lean fish on insulin sensitivity

Author	Subjects	Health	Design	Background diet	Intervention meals	Duration	Results
Dunstan *et al.* (1997)^(^ ^137^ ^)^	*n* 49 37 M 12 F Mean age: 53 (sd 7·7) years Age range: 30–65 years	Type 2 diabetes (no insulin) Mean BMI: 29·6 (sd 3·5) kg/m^2^	RCT, parallel	4 weeks baseline period – normal diet	(1) Fish + moderate exercise (2) Fish and + light exercise (3) No fish + moderate exercise (4) Control (no fish + light exercise) Fish: 7 d/week (turbot, sardines, tuna, salmon)=3·65 g *n*-3 PUFA/d	8 weeks	Fish groups elevated levels of glycated Hb and blood glucose compared with controls
Mori *et al.* (1997)^(^ ^30^ ^)^	*n* 63 42 M 21 F Mean age: 54·1 (sd 1·8) years Age range: 40–70 years	Hypertension Mean BMI: 31·6 (sd 1·05) kg/m^2^	RCT, parallel	4 weeks baseline period – normal diet	(1) Control (weight-maintaining diet) (2) Fish (weight-maintaining diet + fish daily) (3) Weight loss (energy-restricted diet) (4) Fish + weight loss (energy-restricted diet + fish daily) Fish: 7 d/week (turbot, sardines, tuna, salmon)=3·65 g *n*-3 PUFA/d	16 weeks	Fish + weight loss group improved glucose and insulin metabolism
Balfego *et al.* (2016)^(^ ^138^ ^)^	*n* 32 14 M 18 F Mean age: 60·6 (sem 2·1) years Age range: 40–70 years	Type 2 diabetes (no insulin) Mean BMI: 29·7 (sem 0·9) kg/m^2^	RCT, parallel, pilot	2 weeks lead-in period	(1) Control (standard diet, no sardines) (2) Sardine (standard diet + sardines 100 g for 5 d per week)	6 months	Both groups reduced fasting insulin + HOMA-IR to comparable levels (NS group differences). Glycated Hb reduced in the control group
Hallund *et al.* (2010)^(^ ^139^ ^)^	*n* 68 68 M Mean age: 53 (sd 8·3) years Age range: 40–70 years	Healthy Mean BMI: 24·7 (sd 2·3) kg/m^2^	RCT, parallel	Normal diet	(1) Farmed trout – marine diet (2) Farmed trout – vegetable diet (3) Chicken 150 g for 3–4 d per week	8 weeks	NS diet effects on fasting glucose, insulin or HOMA-IR
Zhang *et al.* (2012)^(^ ^141^ ^)^	*n* 126 126 F Mean age: 55·8 (sd 6·7) years Age range: 35–70 years	Hypertriacylglycerolaemia Mean BMI: 26·7 (sd 3·3) kg/m^2^	RCT, parallel	2 weeks run-in (normal diet)	(1) Salmon (2) Herring (3) Pompano (4) Control (meat). Fish/meat: 80 g for 5 d per week	8 weeks	NS diet effects on fasting glucose, insulin or HOMA-IR
Raatz *et al.* (2013)^(^ ^9^ ^)^	*n* 19 8 M 11 F Mean age: 51·6 (sem 1·5) years Age range: 40–65 years	Healthy Mean BMI: 29·2 (sem 0·6) kg/m^2^ BMI range: 25–35 kg/m^2^	RCT, cross-over	Normal diet	Different doses of farmed salmon: 90, 180, 270 g 2 x/week	3 × 4 weeks, 4–8 weeks washout	NS diet effects on fasting glucose, insulin or HOMA-IR
Jacques *et al.* (1992)^(^ ^169^ ^)^	*n* 15 15 F Mean age: 62·5 (sem 0·3) years Age range: 53–79 years	Healthy Postmenopausal Mean BMI: 26 (sem 1) kg/m^2^	RCT, cross-over	Pre-experimental diet (similar to normal diet)	(1) Lean white fish (cod, sole, haddock, halibut, pollock) (2) Non-fish (lean beef, pork, egg, milk) 70–75 % of daily protein replaced with protein from the intervention meals	2 × 4 weeks, 5 weeks washout	Elevated serum sex hormone-binding globulin and higher HDL-cholesterol in lean fish compared with non-fish group (related to improved insulin sensitivity)
Lacaille *et al.* (2000)^(^ ^168^ ^)^	*n* 11 11 M Age range: 19–27 years	Healthy Mean BMI: 24·0 (sem 1·0) kg/m^2^	RCT, cross-over	Pre-experimental diet (similar to normal diet)	(1) Lean fish (cod and sole) (2) Non-fish (lean beef, pork, veal, eggs, skimmed milk, milk products)	2 × 4 weeks, 5 weeks washout	Elevated serum sex hormone-binding globulin and higher HDL-cholesterol in lean fish compared with non-fish group (related to improved insulin sensitivity)
Gascon *et al.* (1996)^(^ ^171^ ^)^	*n* 14 14 F Mean age: 22·4 (sem 0·9) years	Premenopausal Mean BMI: 22 (sem 1·0) kg/m^2^	RCT, cross-over	Pre-experimental diet (similar to normal diet)	(1) Lean fish (cod and sole) (2) Non-fish (lean beef, pork, veal, eggs, skimmed milk, milk products)	2 × 4 weeks, 5 weeks washout	No effects on insulin sensitivity
Aadland *et al.* (2016)^(^ ^76^ ^)^	*n* 20 7 M 13 F Mean age: 50·6 (sem 3·4) years	Healthy Mean BMI: 25·6 (sem 0·7) kg/m^2^	RCT, cross-over	3 weeks run-in period with diet in accordance with Norwegian recommendations	(1) Lean seafood 7 d/week (2) Non-seafood 7 d/week Fish: cod, pollock, saithe, scallops	2 × 4 weeks, 5 weeks washout	Reduced postprandial C-peptide + lactate, no effect on glucose + insulin concentrations in seafood compared with non-seafood group, but reduction in TAG (early markers of improved insulin sensitivity)

M, male; F, female; HOMA-IR, homeostasis model of assessment insulin resistance.

In a randomised parallel pilot trial, thirty-five overweight and obese Spanish T2D patients (not taking insulin or antidiabetic drugs) consumed or did not consume 100 g sardines/d for 5 d per week for 6 months^(^ ^138^ ^)^. Both the control (standard diabetes diet, no sardines) and the sardine group (standard diabetes diet +100 g sardines/d) reduced fasting insulin concentration and homeostasis model of assessment insulin resistance (HOMA-IR) to comparable levels. The blood level of HbA1c was significantly reduced in the control group, and tended to be reduced (*P*=0·08) in the sardine group^(^ ^138^ ^)^.

The effects of daily intake of 150 g rainbow trout farmed either on marine ingredients or with a high content of vegetable ingredients were compared with the daily intake of 150 g chicken fillet in sixty-eight healthy Danish men (aged 40–70 years) in a randomised, parallel 8-week study. No diet effect was found on fasting glucose or insulin concentrations or on HOMA-IR^(^ ^139^ ^)^. Another study compared the effect of eating 125 g farmed salmon daily for 4 weeks with no fish consumption for another 4-week period (control period) in forty-eight healthy Scottish adults (aged 20–55 years). There was no significant effect of daily salmon consumption on fasting glucose or insulin levels, or on HOMA-IR^(^ ^140^ ^)^.

The effect of consuming 80 g oily fish/d five times per week was investigated in a randomised, parallel, 8-week intervention study with 126 adult Chinese women (aged 35–70 years) with baseline high serum TAG levels^(^ ^141^ ^)^. The women ingested Norwegian farmed salmon, herring or Chinese farmed pompano, or a mixture of commonly eaten meats (pork/chicken/beef/lean fish). After 8 weeks, no diet effect was observed on fasting serum glucose and insulin concentrations or on HOMA-IR^(^ ^141^ ^)^.

In an American randomised cross-over study with 4-week diet periods and 4–8 weeks washout periods, nineteen healthy men (*n* 8) and women (*n* 11), aged 40–65 years, consumed 90, 180 or 270 g of farmed salmon two times/week^(^ ^142^ ^)^. After 4-week diet periods, no diet effect was observed on fasting glucose or insulin concentrations or on HOMA-IR. All these trials showed good compliance regarding expected changes in *n*-3 PUFA levels from pre- to post-intervention according to the intervention groups.

## Animal trials with fatty seafood and potential mechanisms of actions

As mentioned above, replacement of fish oil with vegetable oil in salmon feed influences the metabolic effect of the salmon on mice. In particular, the reduced ratio of *n*-3:*n*-6 PUFA in the fish feed, when fish oil was exchanged with soyabean oil, was reflected in the *n*-3:*n*-6 ratio in the salmon, and hence also in the mouse diets. This was associated with increased adiposity, whole-body insulin resistance and hepatic steatosis in mice fed feed containing the farmed salmon^(^ ^66^ ^)^. It was suggested that the low *n*-3:*n*-6 PUFA ratio led to a lower ratio between *n*-3- and *n*-6-derived oxylipins and this might underlie the observed marked metabolic differences. It is not fully elucidated whether a causal link exists between non-alcoholic fatty liver disease (NAFLD) and insulin resistance, but their often co-occurrence and strong links to inflammation are well documented^(^ ^143^ ^,^ ^144^ ^)^.

Marine *n*-3 PUFA efficiently attenuate high-fat diet-induced insulin resistance and NAFLD in rodents, and this may be directly linked to their ability to attenuate obesity development as well as low-grade inflammation^(^ ^145^ ^,^ ^146^ ^)^. It has been suggested that *n*-3 PUFA mediate their anti-inflammatory and insulin-sensitising effect via activation of the GPR120 receptor/FFAR4^(^ ^147^ ^)^. However, conflicting reports suggesting that GPR120/FFAR4 may not be the sole effector have emerged^(^ ^148^ ^,^ ^149^ ^)^, and a number of additional mechanisms probably play a role.

Marine *n*-3 PUFA may replace AA in phospholipids and thereby influence the oxylipin profile. Oxylipins are a broad group of oxygenated polyunsaturated lipids that include the twenty-carbon eicosanoids (PG, leukotrienes and thromboxanes) as well as a number of alcohols, ketones, epoxides and diols. Marine *n*-3 PUFA released from liver phospholipids may also be converted into other *n*-3-derived lipid mediators such as endocannabinoids and eicosanoids that potentially may attenuate the development of both NAFLD and insulin resistance^(^ ^150^ ^,^ ^151^ ^)^. For instance, resolvin D1 has been reported to improve insulin sensitivity in obese diabetic mice, and resolvin E1 and protectin D1 are reported to have both insulin-sensitising and anti-steatotic effects^(^ ^152^ ^,^ ^153^ ^)^. Compared with mice fed salmon with a low content of marine *n*-3 PUFA, increased content of marine *n*-3 PUFA in the salmon led to lower levels of oxylipins derived from AA and higher levels of those derived from EPA and DHA in the liver^(^ ^66^ ^)^. Incorporation of marine *n*-3 PUFA from mouse feed containing salmon into phospholipids in the liver of mice ingesting the feed furthermore leads to reduced substrate availability for endogenous endocannabinoid synthesis^(^ ^26^ ^,^ ^66^ ^)^, representing an additional mechanism by which the *n*-3:*n*-6 ratio PUFA can influence the development of hepatic steatosis and insulin resistance.

Different types of fatty acids have also different capacities to activate Toll-like receptors (TLR), and altered macrophage polarisation is suggested as a mechanism by which marine *n*-3 PUFA alleviate obesity-induced inflammation and insulin resistance^(^ ^154^ ^)^. It has been reported that reduced TLR activation, reduced white adipose tissue inflammation, and improved insulin sensitivity in mice fed marine *n*-3 PUFA, compared with mice fed lard, may in part be attributed to differences in microbiota composition^(^ ^71^ ^)^. The importance of the gut microbiota in the development of insulin resistance is now recognised, but it is not yet clear to what extent the composition and function of the gut microbiota can be modulated by fatty fish.

Using the ‘gold standard’ euglycaemic–hyperinsulinaemic glucose clamp, Lindqvist *et al.* ^(^ ^59^ ^)^ demonstrated that inclusion of herring oil, but not herring mince or herring press juice, into a high-energy diet prevented insulin resistance in rats. This finding indicated that the lipid content of herring was responsible for the beneficial effect. Using the same technique, results from our laboratory demonstrated that adult male rats exposed to crude, but not refined, salmon oil developed insulin resistance^(^ ^155^ ^)^. Fat-soluble environmental pollutants known as POP are present in fatty fish, and there has been growing concern regarding their potential role in the development of T2D^(^ ^17^ ^)^. We have previously observed that POP of marine origin accumulate in adipose tissue concomitant with the development of obesity and insulin resistance in mice fed farmed Atlantic salmon^(^ ^60^ ^)^. However, mice fed a high-fat diet containing both protein and fat from whale were leaner and more insulin sensitive than control casein-fed mice, despite a high accumulation of POP in adipose tissue^(^ ^156^ ^)^. Additionally, when the levels of polychlorinated biphenyls and dichlorodiphenyltrichloroethane (DDT) were reduced by 50 % in salmon fillets by partial replacement of fish oil with vegetable oils in the salmon feed, we observed aggravated insulin resistance and hepatic lipid accumulation^(^ ^64^ ^)^. Further, exposing mice to four of the most abundant POP found in fatty fish, either as single compounds or mixtures, had no effect on obesity development, glucose tolerance or insulin sensitivity^(^ ^157^ ^)^. Still, this study demonstrated that the dietary composition of macronutrients profoundly modulates POP accumulation, an important parameter that needs to be to be included in future studies.

## Acute effects of lean seafood intake on postprandial glucose metabolism

In an acute test meal study, Soucy & LeBlanc^(^ ^158^ ^)^ served healthy Canadian adults either 125 g (*n* 8) or 250 g (*n* 7) of cod fillet or beef in a cross-over design. After the 125 g meals, plasma insulin concentration, concentrations of several amino acids and total amino acids, and carbohydrate oxidation were higher 180 min after intake of beef as compared with intake of the cod meal. These differences were not observed after the 250 g meal^(^ ^158^ ^)^. As lean seafood contains more water than terrestrial meat, consuming fillets of the same weight will result in a higher protein intake from the terrestrial meat. Thus, Soucy & LeBlanc^(^ ^159^ ^)^ performed another study in healthy adults, in which they compared either 43 g protein from cod fillet (250 g cod) or beef (195 g beef), or 250 g of cod or beef fillet (equal to 43 g cod protein and 55 g beef protein)^(^ ^159^ ^)^. At both protein doses, the postprandial amino acid response and oxygen consumption were higher after the beef meals as compared with after the cod fillet intake, indicating differences in energy metabolism following the two meals. No significant difference was found for postprandial insulin concentration. In both studies, the meals consisted of only cod fillet or beef (i.e. no carbohydrates) and the postprandial plasma glucose remained at the pre-meal levels^(^ ^158^ ^,^ ^159^ ^)^.

Recently, we compared the postprandial glucose metabolism after consumption of complete test meals (2012 kJ; 25·5, 33·5 and 41 %E from protein, fat and carbohydrate, respectively) with either 115·5 g cod fillet or 100 g veal in overweight adults (*n* 21). We observed no difference in postprandial concentrations of glucose, lactate, insulin or C-peptide following ingestion of meals with cod or veal^(^ ^87^ ^)^. The acute meal effect of cod has also been compared with non-meat protein sources. In one test meal study, healthy women (*n* 17) received three test meals (2300 kJ; 33, 26 and 41 %E from protein, fat and carbohydrate, respectively) with 45 g protein as cod fillet, cottage cheese (milk protein), or soya protein isolate. Ingestion of the cod protein meal resulted in higher postprandial AUC for glucose (0–120 min), and lower serum insulin:glucose and insulin:C-peptide ratios, as compared with the cottage cheese meal, suggesting that different protein sources affect glucose and insulin metabolism differently^(^ ^160^ ^)^. In obese, non-diabetic adults (*n* 11) ingestion of high-energy, high-fat (about 4920 kJ; 15, 66 and 19 %E from protein, fat and carbohydrate, respectively) liquid test meals resulted in lower postprandial glucose and higher insulin concentrations after consumption of 45 g whey isolate as compared with after intake of 45 g cod fillet, gluten or casein^(^ ^161^ ^)^. Thus, even though the acute postprandial effect of test meals containing cod on glucose metabolism has been tested under varying settings, the general picture is that minor differences in postprandial concentrations of insulin and glucose are found comparing red meats with cod, whereas milk proteins, in particular whey, induce a higher postprandial insulin response leading to reduced postprandial glucose concentrations as compared with the intake of cod.

After a meal, gut incretin hormones are secreted and promote postprandial insulin secretion and regulate glucagon secretion, and the interest in selective glucagon-like peptide-1 (GLP-1) receptor agonists for the treatment of T2D and obesity has increased^(^ ^162^ ^)^. Secretion of GLP-1 from the intestine, together with secretion of cholecystokinin (CCK) from duodenal cells, will also participate in mediating satiety signals. High-protein diets are suggested to increase satiety, partly by inducing secretion of incretins, and different protein sources may affect secretion of GLP-1 and CCK differently^(^ ^163^ ^,^ ^164^ ^)^. As mentioned above, whey protein intake induces a high postprandial insulin response, probably due to a rapid increase in postprandial amino acids, including branched-chain amino acids, known to induce insulin secretion^(^ ^165^ ^)^. Whey is also known to induce postprandial increased levels of GLP-1 and gastric inhibitory peptide (GIP). Furthermore, amino acids and peptides from whey digestion are suggested to inhibit dipeptidyl peptidase 4, thereby prolonging signalling through GLP-1 and GIP by preventing their degradation^(^ ^165^ ^)^. Protein from cod is reported to be less effective than pea protein and wheat protein in increasing CCK and GLP-1 release in human duodenal tissue^(^ ^166^ ^)^. Whether seafood protein is more effective than proteins from terrestrial animals to induce secretion of incretins is to our knowledge not known, but a fish protein hydrolysate has been reported to stimulate secretion of both GLP-1 and CCK^(^ ^167^ ^)^. Slightly overweight (25 kg/m^2^ ≤ BMI < 30 kg/m^2^) subjects (*n* 109) between 18 and 55 years were subjected to a mild hypoenergetic (−300 kcal/d; –1255 kJ/d) diet and randomised to receive 1·4 or 2·8 g fish protein hydrolysate from blue whiting or whey protein as placebo for 90 d. The serum levels of CCK and GLP-1 were measured after 45 and 90 d. Compared with placebo, both the 1·4 and 2·8 g fish protein hydrolysate dose increased CCK and GLP-1 levels at both time points. This was accompanied with reduced body weight, fat mass, as well as reduced waist, thigh and hip circumferences^(^ ^167^ ^)^.

## Frequent high lean seafood intake and effects on insulin sensitivity

The effect of frequent lean seafood intake on glucose regulation and insulin sensitivity has also been studied (Table 2). In randomised controlled 4-week intervention studies with a cross-over design, a high proportion (69–75 %) of the daily protein intake (18–20 %E protein) was given as either lean seafood or as lean non-seafood sources (primarily lean meats). From these studies, it was shown in Canadian men and postmenopausal women that daily inclusion of lean fish fillets for 28 d, at the expense of other animal protein sources, resulted in elevated serum sex hormone-binding globulin^(^ ^168^ ^,^ ^169^ ^)^ as well as higher HDL_2_-cholesterol concentrations^(^ ^168^ ^,^ ^170^ ^)^. Under similar study settings, these differences were not found in premenopausal women in whom rather a decrease in serum TAG level was observed^(^ ^171^ ^)^. As elevated serum concentrations of HDL_2_-cholesterol and sex hormone-binding globulin and reduced serum TAG levels are associated with improved insulin sensitivity^(^ ^172^ ^,^ ^173^ ^)^, these observations support that a frequent high intake of lean fish, as compared with frequent high lean meat intake, might improve insulin sensitivity in adults. In line with these observations, improved insulin sensitivity was confirmed by the hyperinsulinaemic clamp technique in Canadian men and women who were insulin resistant at start of the intervention, but had significant improvement in insulin sensitivity by ingesting cod daily for 4 weeks (58–68 % of daily protein intake) as compared with intake of a lean meat-based diet^(^ ^174^ ^)^.

We have shown in Norwegian healthy men and women that a high (60 % of total protein) daily intake of lean seafood for 4 weeks reduced postprandial concentrations of C-peptide and lactate, without affecting glucose or insulin concentrations as compared with an equal amount of non-seafood diet (mainly lean meats)^(^ ^76^ ^)^. Moreover, we also found reductions in fasting and postprandial concentrations of TAG, medium-sized VLDL particles and the TAG:HDL-cholesterol ratio^(^ ^76^ ^,^ ^77^ ^)^. As elevated concentrations of C-peptide^(^ ^175^ ^)^ and lactate^(^ ^176^ ^,^ ^177^ ^)^, as well as an increased TAG:HDL-cholesterol ratio^(^ ^178^ ^,^ ^179^ ^)^, may be useful predictors of dysregulated glucose metabolism and/or early markers of insulin resistance, our data are in line with the above-mentioned observations that a frequent high intake of lean seafood may prevent, and possibly reverse, insulin resistance relative to a frequent high intake of meat-based diets.

## Animal trials with lean seafood and potential mechanisms of actions

From studies with rats, it has been shown that both cod and soya protein feeding resulted in reduced fasting and postprandial glucose and insulin responses, as well as improved peripheral insulin sensitivity, relative to rats fed the milk protein casein^(^ ^180^ ^)^. In follow-up studies with a high-fat, high-sucrose diet, cod protein feeding, as compared with soya protein and casein feeding, prevented rats from developing skeletal muscle insulin resistance^(^ ^181^ ^)^ by normalising skeletal muscle insulin-stimulated phosphoinositide 3-kinase activity and downstream protein kinase B (Akt) signalling and by improving translocation of GLUT4 to cell-surface membranes^(^ ^182^ ^)^. The above-mentioned rat studies^(^ ^180^ ^–^ ^182^ ^)^ were performed with diethyl ether-extracted cod fillets to remove the small amount of endogenous fat present in the cod fillets. It is therefore tempting to speculate that the cod protein fraction, or molecules present in the protein fraction, may prevent the development of insulin resistance and T2D.

This protective effect may not be restricted to cod as it has also been demonstrated that protein from sardines attenuates fructose-induced insulin resistance, obesity and accompanying inflammation in adipose tissue in rats^(^ ^183^ ^)^. However, in an experiment where rats were fed hydrolysed proteins from either bonito, herring, mackerel or salmon in a high-fat, high-sucrose diet, neither of the hydrolysed fish protein sources influenced glucose tolerance compared with casein^(^ ^184^ ^)^. Still, using the hyperinsulinaemic–euglycaemic clamp technique, it was demonstrated that hydrolysed proteins from salmon prevented high-fat, high-sucrose-induced whole-body insulin resistance. Further, compared with casein-fed rats, rats fed hydrolysed salmon as well as hydrolysed bonito, herring and mackerel had lower expression of inflammatory markers in white adipose tissue. Of note, however, only hydrolysed salmon protein led to reduced white adipose tissue mass^(^ ^184^ ^)^.

Compared with mice fed lean seafood, we have observed impaired glucose tolerance and mild insulin resistance in mice fed Western diets with lean meat from terrestrial animals^(^ ^99^ ^)^. The observed changes in microbiota tyrosine and phenylalanine metabolism might be of relevance, as increased fasting plasma concentrations of the aromatic amino acids are associated with the development of insulin resistance and T2D^(^ ^185^ ^)^. Further, an increased capacity for production of branched-chain amino acids (BCAA) in the gut microbiota and increased plasma levels of BCAA have also been shown to be associated with insulin resistance^(^ ^186^ ^)^. Still, the link between the gut microbiota, circulating amino acids and the development of insulin resistance is far from understood.

Evidently, reduced fat accumulation and thereby reduced infiltration of pro-inflammatory macrophages may, at least in part, explain why the development of insulin resistance is attenuated by inclusion of some dietary fish proteins. However, other mechanisms may also be involved. In rats, at least cod protein appears to prevent the development of insulin resistance in muscle independent of adipose tissue mass^(^ ^180^ ^,^ ^182^ ^)^, and insulin-stimulated glucose uptake has been stimulated in L6 myocytes exposed to a cod-derived amino acid mixture^(^ ^181^ ^)^, indicating a direct effect of these amino acids on glucose uptake activated by insulin. Further, a higher dietary content of the amino acids arginine, glycine, taurine and lysine as found in cod protein has previously been associated with anti-inflammatory effects in rats^(^ ^187^ ^)^.

## Studies comparing frequent intake of lean and fatty fish on regulation of glucose metabolism

A few studies have compared the intake of lean fish *v.* fatty fish in relation to glucose metabolism. One RCT investigated the effect of consuming two portions/week of white fish (cod, prawns, fishcakes, canned tuna) or fatty fish (salmon, mackerel, salmon fishcakes, canned salmon) for 24 weeks in overweight and obese UK men and women aged 35–65 years^(^ ^188^ ^)^. Compliance was evaluated and changes in fatty acid status correlated well with dietary intake. There was no significant diet effect on fasting plasma glucose and insulin concentrations, or on plasma measures following a 75 g oral glucose tolerance test^(^ ^188^ ^)^. In a Swedish randomised study with cross-over design, eight women and eight men, aged 37–75 years and diagnosed with T2D, consumed daily diets with lean or fatty fish for two consecutive 3·5-week diet periods^(^ ^189^ ^)^. Compliance was accounted for, and linoleic acid measured in plasma increased following intake of the *n*-6 diet, and plasma *n*-3 PUFA increased following intake of the fatty fish diet. The participants did not receive insulin treatment, but thirteen of the sixteen participants were treated with oral antidiabetic drugs. Following the lean fish diet period, fasting blood glucose was reduced, and fasting serum C-peptide tended to be reduced, as compared with after the fatty fish diet period. Moreover, following a breakfast meal, the postprandial glucose AUC was reduced, and the insulin AUC was increased after the lean fish period, as compared with after the fatty fish diet period^(^ ^189^ ^)^. In a Norwegian parallel-arm pilot study with free-living young subjects (20–35 years of age) who were supplied with 750 g/week (150 g portions 5 d/week), the dietary effects of cod (*n* 13), farmed salmon (*n* 14) or chicken fillet without skin (*n* 11) were compared for 4 weeks of intervention^(^ ^190^ ^)^. No significant differences were found between diet groups on fasting and postprandial glucose, insulin or C-peptide concentrations following ingestion of a standardised breakfast meal (1905 kJ; 8 g fat, 8 g protein and 85 g carbohydrates) at baseline and after 4 weeks of intervention. A similar study design was used in a second study by Helland *et al.* ^(^ ^191^ ^)^ with 750 g fish/week (150 g portions 5 d/week) administered to free-living, healthy, overweight Norwegian adults (18–69 years). The trial was performed over a period of 8 weeks with three parallel intervention arms including a lean fish group (*n* 22; cod), a fatty fish group (*n* 23; farmed salmon) and a control group (*n* 20; no fish). The results from the primary outcome measures, serum postprandial glucose concentration, showed that high intake of fatty fish, but not lean fish, reduced postprandial glucose at 90 and 120 min after a standardised test meal. The postprandial C-peptide concentration was significantly reduced at 120 min after the test meal in the fatty fish group only. Analyses of fatty acids composition showed good compliance.

## Seafood intake and C-reactive protein

Low-grade inflammation may be one underlying mechanism of metabolic disease, and C-reactive protein (CRP) is an acute-phase protein whose elevated circulating level has been associated with poor glycaemic control^(^ ^192^ ^,^ ^193^ ^)^, development of T2D^(^ ^194^ ^,^ ^195^ ^)^ and mortality in T2D^(^ ^196^ ^)^. Two intervention studies have reported reduced CRP levels after seafood intake, one cross-over study in insulin-resistant subjects comparing 4-week diet periods with cod or non-fish diets^(^ ^197^ ^)^, and one multi-centre, parallel, randomised controlled intervention study in which participants received dietary advice alone or in combination with 150 g fish twice weekly; 300 g salmon/week or 300 g cod/week for 6 months^(^ ^198^ ^)^. Moreover, in a Greek cross-sectional study, reduced levels of inflammatory markers were reported in individuals consuming >300 g of fish/week as compared with non-fish consumers^(^ ^199^ ^)^.

By contrast, in other intervention studies with fatty fish such as herring^(^ ^141^ ^,^ ^200^ ^)^, sardines^(^ ^138^ ^)^, farmed salmon^(^ ^140^ ^–^ ^142^ ^)^, farmed trout^(^ ^139^ ^)^, a mixture of fatty fish species^(^ ^201^ ^)^ or fatty (farmed salmon) and lean (cod)^(^ ^190^ ^)^ no changes in CRP concentrations were observed.

A CRP concentration >3 mg/l is associated with an increased OR for developing T2D^(^ ^194^ ^)^. In older Australians consuming either a diet rich in fatty fish or a non-fish diet, a secondary analysis revealed that the participants with baseline CRP levels >3 mg/l increased their CRP values after the meat-based non-fish diet as compared with after the fatty fish diet^(^ ^201^ ^)^. Thus, these results indicate that a high fish intake may in some cases, but not all, be beneficial to reduce CRP levels in subjects.

## Seafood intake and adiponectin

Adiponectin is a signalling molecule secreted from adipocytes that have anti-inflammatory and insulin-sensitising properties. Low circulating adiponectin levels are a risk marker of incident prediabetes^(^ ^202^ ^)^, and higher adiponectin levels are associated with reduced risk of T2D^(^ ^203^ ^)^. The adiponectin level is increased by activation of PPAR-γ, which is, among others, activated by PUFA^(^ ^204^ ^)^. Intervention studies with fatty fish rich in marine *n*-3 PUFA, such as farmed salmon^(^ ^140^ ^,^ ^141^ ^,^ ^190^ ^)^, herring and farmed Chinese pompano^(^ ^141^ ^)^, and sardines^(^ ^138^ ^)^ consistently increased adiponectin concentration. In contrast, intervention studies with lean seafood, less rich in marine *n*-3 PUFA, did not elevate the adiponectin level^(^ ^76^ ^,^ ^190^ ^,^ ^197^ ^)^. Thus, based on available data, only intake of fatty seafood is associated with an increased adiponectin level.

## Future perspectives for intervention studies

A common drawback in relation to most randomised clinical trials and dietary intervention trials in general is the study design with primary endpoints and outcomes in relation to all participants in each arm of the study, with no stratification between responders and non-responders. While the importance of personalised or stratified medical treatment now receives considerable attention and large programmes are being pursued in many countries, the intervention studies discussed in the present review did not consider stratification in relation to possible responders and non-responders in the examined groups except for differences between males and females. While genetic background including specific single penetrant polymorphisms or mutations for long has been known to profoundly affect the response to intake of certain dietary components such as phenylalanine and lactose, the importance of the gut microbiota in relation to metabolic responses to dietary intake has only recently been convincingly documented^(^ ^205^ ^)^. Until now official dietary advice has also in general been based on the belief that one size fits all, neglecting the inter-individual variabilities in dietary responses. A seminal article published in 2015 demonstrated the power of personalised dietary recommendation based on the composition and functional potential of the gut microbiota^(^ ^39^ ^)^. Since then it has been demonstrated how dietary metabolic responses in relation to risk factors for CVD and T2D show inter-individual variability, and that responses to certain changes in lifestyle vary between individuals^(^ ^205^ ^)^. All this calls for a re-evaluation of how to design and interpret intervention studies, which in the future should combine personalised information on genetics, epigenetics, metabolomics and metagenomics. This also implies the use of big data, the development of novel machine learning algorithms, and eventually the use of artificial intelligence. Thus, it is possible that beneficial effects in response to previous dietary intervention trials have been blurred by the study design, and that reanalysis of available data using stratification according to responders/non-responders would reveal more interesting beneficial effects of specific diets to a subgroup of individuals taking part in the intervention trials.

## Conclusion

Overweight and obesity development is for most individuals the result of years of positive energy balance. A growing body of evidence from intervention trials and animal studies suggests that a frequent intake of lean seafood, as compared with intake of terrestrial meats, reduces energy intake typically in the range of 4–9 %, a reduction sufficient to prevent a positive energy balance and obesity. The data from lean seafood intake are largely in agreement with observational data.

Regarding the intake of fatty fish, observational data from one study indicate that intake of fatty fish was associated with increased body weight. Data from intervention trials or animal studies do not support the observational data linking a high fatty fish intake to body weight gain. During weight reduction, i.e. energy restriction, intake of both lean and fatty seafood may increase body-weight loss. Intake of marine *n*-3 PUFA is probably of importance for reduced fat mass, possibly through *n*-3 PUFA-derived signalling molecules like endocannabinoids and oxylipins.

As with obesity, the development of insulin resistance and T2D normally occurs over many years. The majority of data, both from interventions and from animal studies, suggest that a frequent intake of lean seafood as compared with intake of terrestrial meats reduces both fasting and postprandial risk markers of insulin resistance, as well as improving insulin sensitivity in already insulin-resistant adults. The exception is shellfish and fried lean fish, the intake of which is associated with impaired glycaemic control. In healthy subjects, a high intake of fatty fish appears to have neutral effect on fasting markers of insulin sensitivity, but intake of fatty fish has been reported to improve postprandial glycaemic control. A high intake of fatty fish in subjects with diabetes or hypertension may impair glycaemic control, unless combined with exercise or weight reduction.

Intake of fatty fish increases plasma concentration of the insulin-sensitising adipocyte-derived signal molecule adiponectin. As compared with a high meat intake, high intake of seafood has been reported to reduce the hepatic acute-phase protein CRP plasma level in some, but not all studies.

Further studies are needed to confirm the dietary effects on energy intake, obesity and insulin resistance. In addition, future studies should be designed to elucidate the potential contribution of trace elements, vitamins and undesirables present in seafood. Finally, we argue that stratification into responders and non-responders in randomised clinical trials may improve the understanding of health effects from intake of seafood in future trials.
